# Incidence, associated factors and prognostic association of acute kidney injury after TIPS: a multicenter retrospective study

**DOI:** 10.1080/0886022X.2026.2650577

**Published:** 2026-04-13

**Authors:** Jun Shang, Pingwei Cheng, Han Cao, Jie Wu, Delei Cheng, Jinchang Xiao, Hao Xu, Bo Jiang, Qi Cao, Chunze Zhou, Ruifeng Wang

**Affiliations:** a Department of Nephrology, The Second Affiliated Hospital of Anhui Medical University, Hefei, China; b Anhui Medical University, Hefei, China; c Inflammation and Immune Mediated Diseases Laboratory of Anhui Province, Hefei, China; d Department of Interventional Radiology, The First Affiliated Hospital, University of Science and Technology of China, Hefei, China; e Affiliated Hospital of Xuzhou Medical University, Xuzhou, China; f Department of Interventional Radiology, The Second Hospital of Anhui Medical University, Hefei, China; g Centre for Transplant and Renal Research, Westmead Institute for Medical Research, The University of Sydney, Sydney, NSW, Australia

**Keywords:** decompensated cirrhosis, prognosis, portal hypertension, acute kidney injury, transjugular intrahepatic portosystemic shunt

## Abstract

This study investigated the incidence, risk factors, and prognostic implications of acute kidney injury (AKI) after transjugular intrahepatic portosystemic shunt (TIPS). This multicenter retrospective study included patients who underwent TIPS at three hospitals in China. AKI risk factors were identified using multivariate logistic regression in the overall cohort. Propensity score matching was performed to balance baseline covariates. Survival differences were assessed using the Kaplan-Meier method, and the association between AKI and mortality was evaluated using stratified Cox regression models. A total of 995 patients were included, of whom 4.92% developed postoperative AKI. Multivariable analysis identified older age (OR: 1.07, 95% CI: 1.04–1.10), higher preoperative neutrophil percentage (OR: 1.05, 95% CI: 1.02–1.08), elevated preoperative creatinine (OR: 1.01 [per μmol/L], 95% CI: 1.00–1.01), and an increased Child-Pugh score (OR: 1.27, 95% CI: 1.01–1.59) as independent risk factors for AKI. AKI was strongly associated with increased overall mortality (*p* < 0.001). In the propensity score-matched cohort, postoperative AKI remained an independent predictor of worse long-term survival (HR: 4.09, 95% CI: 1.97–8.50). In conclusion, age, preoperative neutrophil percentage, creatinine level, and Child-Pugh score were independent risk factors for AKI following TIPS, and the development of AKI is strongly associated with a poor prognosis in these patients.

## Introduction

Transjugular intrahepatic portosystemic shunt (TIPS) is a minimally invasive, well-established procedure for managing complications of cirrhotic portal hypertension, such as variceal bleeding and refractory ascites [1,2]. While effective, TIPS is associated with serious complications, including hepatic encephalopathy and hepatic decompensation [3,4]. Acute kidney injury (AKI) is a particularly concerning complication linked to significantly increased mortality and healthcare costs in these patients [5].

Although TIPS can improve renal hemodynamics in selected patients, peri-procedural AKI remains a critical clinical challenge. A retrospective analysis of the National Inpatient Sample (NIS) database demonstrated that advanced chronic kidney disease (CKD) is an independent risk factor for post-TIPS AKI and mortality [6]. Another study suggested intravenous contrast administration during TIPS may be associated with a higher AKI risk, particularly in patients with preexisting renal dysfunction [7]. However, the existing evidence remains limited by single-center designs, inconsistent AKI definitions, and a lack of comprehensive risk assessment that integrates patient demographics, inflammatory status, and hepatic reserve [8,9]. Consequently, the true incidence, precise risk profile, and prognostic impact of AKI following TIPS are not well characterized, highlighting the need for robust, multicenter investigations.

In this large, multicenter retrospective study, we aimed to investigate the incidence, identify independent perioperative risk factors, and evaluate the survival impact of AKI in patients undergoing TIPS, using the standardized diagnostic criteria proposed by the International Club of Ascites (ICA) [10].

## Materials and methods

### Patients

This study retrospectively included adult patients (≥18 years old) diagnosed with decompensated cirrhosis complicated by variceal bleeding or recurrent/refractory ascites who underwent TIPS treatment, based on liver function tests, imaging, and hepatic puncture biopsy, at the Second Affiliated Hospital of Anhui Medical University, the First Affiliated Hospital of the University of Science and Technology of China, and the First Affiliated Hospital of Xuzhou Medical University between January 2015 and December 2023.

This retrospective study was approved by the Ethics Committee of the Second Affiliated Hospital of Anhui Medical University (Approval No: YX2024-215) and conducted in accordance with the Declaration of Helsinki. Due to the retrospective nature of this multicenter study, the requirement for informed consent was addressed as follows: surviving participants were contacted and provided written informed consent after being fully informed of the study. For patients who were deceased or could not be reached despite reasonable efforts, the Ethics Committee granted a waiver of informed consent, and only fully anonymized data were used with strict privacy protection.

The inclusion criteria were as follows: (1) age ≥ 18 years and (2) decompensated cirrhosis complicated by variceal bleeding or recurrent/refractory ascites who underwent TIPS. The diagnosis of cirrhosis was established based on consideration of etiology, medical history, clinical manifestations, complications, treatment process, laboratory tests, imaging studies, and histological examinations. The exclusion criteria were as follows: (1) preexisting AKI before the procedure, (2) hemodialysis or peritoneal dialysis prior to the procedure, and (3) incomplete baseline data. The diagnosis and staging of AKI were based on the 2015 ICA criteria, defined as an increase in serum creatinine (Scr) of ≥26.5 μmol/L (0.3 mg/dL) within 48 h or an increase of ≥50% from baseline within 7 days from completion of the TIPS procedure.

### Data collection

Baseline clinical data, including age, sex, time of surgery, and comorbidities, were collected from all patients who underwent TIPS by reviewing their electronic medical records. Diabetes mellitus was defined as a documented history of diabetes or use of glucose-lowering medications. Use of diuretics was recorded based on medication history at hospital admission. CKD status was assessed using the baseline estimated glomerular filtration rate (eGFR), calculated with the CKD-EPI 2009 equation [11]. A binary variable “CKD” was defined as eGFR < 60 mL/min/1.73 m^2^ at hospital admission [12]. Additionally, laboratory test results related to preoperative and postoperative TIPS were collected, including levels of albumin, leukocytes, creatinine, aspartate aminotransferase (AST), international normalized ratio (INR), alanine aminotransferase (ALT), serum total bilirubin, platelet count, neutrophil count, lymphocyte count, and prothrombin time (PT). Baseline Scr was defined as the most recent preoperative value available within 24 h prior to TIPS. To rigorously apply the ICA criteria and avoid reliance on a single postoperative value, all available Scr measurements within the 0–48 h and 0–7 day windows were retrieved. The maximum Scr value within each window was used for comparison with baseline to adjudicate AKI. Autoimmune and autoimmune-associated cholestatic liver diseases were consolidated into a single ‘autoimmune’ category for analysis due to their shared immune-mediated pathophysiology. All-cause mortality was evaluated as the primary outcome through telephone follow-up conducted by specialized personnel and by reviewing the patients’ medical records. Mortality rates at 30-day, 90-day, 1-year, 2-year, and 3-year intervals were recorded as secondary outcomes. Follow-up was defined from the date of TIPS to June 2024, with earlier censoring at death or liver transplantation. Data were compiled and analyzed after follow-up was completed.

### MELD score calculation

The Model for End-Stage Liver Disease (MELD) score was calculated using the standard formula: MELD = 3.78 × ln[serum bilirubin (mg/dL)] + 11.2 × ln[INR] + 9.57 × ln[serum creatinine (mg/dL)] + 6.43, where ln denotes the natural logarithm. For patients receiving renal replacement therapy, the serum creatinine value was set to 4.0 mg/dL. The formula for calculating the MELD score is provided in the Supplementary Material.

### Definition and grading of ascites

The severity of ascites was assessed at hospital admission prior to the TIPS procedure. Grading was based on physical examination and abdominal ultrasonography, in accordance with the clinical practice guidelines proposed by the International Club of Ascites [13]. Ascites was categorized as follows: Grade 1 (Mild): Ascites detectable only by ultrasonography. Grade 2 (Moderate): Ascites manifesting as moderate, symmetrical abdominal distension, confirmed by the presence of shifting dullness on physical examination. Grade 3 (Severe/Massive): Ascites causing gross abdominal distension and tension, often accompanied by dyspnea or other symptoms of abdominal compartment syndrome. For the purpose of statistical analysis in this study, grades 2 and 3 were combined into a single category (“moderate to massive”) as presented in Table 1.

**Table 1.** Baseline characteristics of the study Population.

Baseline variables	Overall	AKI group	Non-AKI group	*p* value
	(*n* = 995)	(*n* = 49)	(*n* = 946)	
Age, yr	55.20 ± 11.40	62.88 ± 11.08	54.76 ± 11.26	<0.001
Male	701 (70.45)	32 (65.30)	669 (70.70)	0.42
Ascites:				0.14
0 (None/Mild)	700 (70.40)	31 (63.32)	669 (70.71)	
1 (Moderate/Massive)	195 (19.59)	18 (36.73)	177 (18.71)	
Comorbid hypertension	103 (10.35)	7 (14.28)	96 (10.15)	0.35
Use of diuretics	34 (3.42)	3 (6.12)	31 (3.28)	0.03
Furosemide	17 (1.71)	2 (4.08)	15 (1.58)	
Spironolactone	17 (1.71)	1 (2.04)	16 (1.69)	
Etiology of cirrhosis:				0.75
Viral	601 (60.40)	26 (53.06)	575 (60.78)	
Alcoholic	76 (7.64)	4 (8.16)	72 (7.61)	
Autoimmune	46 (4.62)	3 (6.12)	43(4.54)	
Other	272 (27.34)	16 (32.65)	256 (27.06)	
Comorbid CKD	74 (7.44)	18 (36.73)	56 (5.92)	<0.001
Comorbid diabetes	157 (15.78)	14 (28.57)	143 (15.12)	0.01
White blood cells (×10⁹/L)	3.62 (2.30, 5.53)	5.41 (3.48, 7.91)	3.48 (2.27, 5.38)	<0.001
Neutrophils (×10⁹/L)	2.21 (1.36, 3.81)	3.97 (2.66, 5.30)	2.11 (1.34, 3.70)	<0.001
Lymphocytes (×10⁹/L)	0.69 (0.42, 1.06)	0.72 (0.37, 1.40)	0.69 (0.44, 1.04)	0.83
Neutrophil percentage (%)	65.62 ± 14.25	73.65 ± 12.02	65.21 ± 14.24	<0.001
NLR	3.28 (2.03, 5.44)	5.30 (3.66, 8.01)	3.18 (1.98, 5.28)	<0.001
Hemoglobin (g/L)	80.00 (65.00,101.00)	80.00 (62.00,96.00)	80.00 (65.00, 101.00)	0.32
Platelets (×10⁹/L)	67.00 (46.50, 106.50)	69.00 (48.00,117.50)	67.00 (46.00, 106.00)	0.66
ALT (U/L)	22.00 (15.00,35.45)	23.00 (15.50,44.10)	22.00 (15.00,34.50)	0.25
AST (U/L)	31.00 (22.00,46.00)	33.10 (24.00,46.50)	30.75 (22.00,46.00)	0.18
TBIL (μmol/L)	21.40 (14.10,32.90)	22.30 (13.90,33.50)	21.30 (14.12,32.75)	0.66
ALB (g/L)	32.81 ± 10.53	30.21 ± 5.39	32.95 ± 10.71	0.08
Blood urea nitrogen (mmol/L)	6.00 (4.38,7.90)	10.48 (7.29,20.40)	5.90 (4.30,7.88)	<0.001
Creatinine (μmol/L)	63.00 (52.00,78.00)	90.00 (52.00,142.10)	63.00 (52.00,75.00)	<0.001
PT (s)	14.90 (13.30,16.75)	15.80 (13.80,19.20)	14.85 (13.30,16.60)	0.04
TT (s)	17.70 (16.30,18.90)	18.05 (16.62,20.23)	17.60 (16.28,18.80)	0.05
INR	1.26 (1.12,1.43)	1.29 (1.18,1.56)	1.26 (1.12,1.43)	0.09
SBP (mmHg)	115.61 ± 15.45	116.67 ± 14.69	114.55 ± 16.33	0.58
DBP (mmHg)	68.53 ± 12.48	69.36 ± 12.29	67.70 ± 12.80	0.59
Preoperative PPG (mmHg)	24.80 ± 5.30	25.85 ± 3.70	22.60 ± 5.30	0.03
Postoperative PPG (mmHg)	11.10 ± 3.10	11.45 ± 3.10	10.64 ± 3.20	0.29
Treatment Center				0.69
Center 1 (*n* = 246)	246 (24.72)	13 (26.53)	233 (24.63)	
Center 2 (*n* = 736)	736 (73.97)	36 (73.47)	700 (74.00)	
Center 3 (*n* = 13)	13 (1.31)	0 (0.00)	13 (1.37)	

Abbreviations: AKI, acute kidney injury; ALB, albumin; ALT, alanine aminotransferase; AST, aspartate aminotransferase; CKD, chronic kidney disease; DBP, diastolic blood pressure; INR, international normalized ratio; NLR, neutrophil-to-lymphocyte ratio; PPG, portal venous pressure gradient; PT, prothrombin time; SBP, systolic blood pressure; TBIL, total bilirubin; TT, thrombin time. Continuous variables are expressed as mean ± standard deviation (SD) or median (interquartile range, IQR), as appropriate. Categorical variables are expressed as number (percentage, %).

### TIPS procedure

All TIPS implantation procedures were conducted by experienced and specially trained interventional radiologists at each center. During the procedure, most patients received local anesthesia. The right internal jugular vein was punctured (a few patients had punctured the left internal jugular vein). The hepatic vein was cannulated through the caval vein using a RUPS-100 puncture device. The portal vein was punctured under fluoroscopy to establish a direct hepatic vein-portal vein channel. Subsequently, the thick varicose veins were embolized, and a hepatic vein-portal vein shunt channel was created by balloon dilatation (6–8 mm) and implantation of a PTFE overmolded stent. The Portosystemic Pressure Gradient (PPG) was measured before and after shunt establishment. The target PPG value was less than 12 mmHg or at least 20% lower than the baseline. Nonionic isotonic contrast media (ioversol, iohexol, or iodixanol) at an iodine concentration of 300–320 mg I/mL were uniformly used in all TIPS procedures. The dosage of contrast media was individually adjusted according to the patient’s body weight and the complexity of the operation.

### Statistical analysis

All statistical analyses were performed using SPSS version 26.0 and R version 4.3.1, with two-tailed tests. Normally distributed continuous data are presented as mean ± standard deviation, and intergroup comparisons were conducted using independent *t*-tests. Non-normally distributed data are expressed as median (interquartile range), and inter-group comparisons were performed using the Mann-Whitney *U* test. Categorical data are presented as frequency and percentage (%), and inter-group comparisons were made using the chi-square test (*χ*^2^). For risk factor analysis of AKI, univariate logistic regression was used, and variables with *p* < 0.1 were included in multivariable logistic regression to identify independent risk factors. Multicollinearity was assessed using variance inflation factors (VIF). Variables with VIF > 5 were excluded from the multivariate model.

The primary multivariable logistic regression model (Model 1) included the following continuous variables: age, preoperative neutrophil percentage, preoperative creatinine level, and Child-Pugh score. All regression models were used to assess the robustness of our findings. We performed sensitivity analyses using the following alternative models, as detailed in Table S1: Model 2: Model 1 with additional adjustment for diabetes and hypertension. Model 3: Model 1 with the MELD score replacing the Child-Pugh score. To minimize confounding in the survival analysis, we performed propensity score matching (PSM) and selected optimal matching with a 1:2 ratio. Matching was based on the following covariates: age, sex, Child-Pugh score, preoperative creatinine, hemoglobin, leukocyte count, cirrhosis etiology, presence of hepatoma, diabetes, hypertension, and albumin. Covariate balance was assessed using standardized mean differences (SMD) [14,15]. The Kaplan-Meier survival curves were used to visualize the overall survival of the AKI group and the non-AKI group. Before matching, the log-rank test was applied to compare the survival differences between the two groups. After matching, to fully account for the paired structure, the stratified log-rank test was adopted for the comparison of survival differences, where each matched pair was treated as an independent stratum. Consistently, we employed a stratified Cox proportional hazards regression model, with each matched set defining a separate stratum. To assess the robustness of the association between AKI and mortality when accounting for residual confounding, a sensitivity analysis was performed in the matched cohort by adding all covariates that remained imbalanced after matching (creatinine, diabetes, hypertension) to the primary stratified Cox model (Table S5, Model B). Furthermore, to assess the robustness of the association between AKI and mortality when excluding the center with no AKI cases, we repeated the primary multivariable logistic and Cox regression analyses after excluding data from Center 3. The results of these analyses are presented in Tables S2 and S3. Statistical significance was set at *p* < 0.05.

**Table 2.** Univariable logistic regression analysis of risk factors for post-TIPS AKI.

Variables	OR	95% CI	*p* value
Age, yr	1.07	1.04–1.10	<0.001
Male	1.28	0.70–2.35	0.42
Etiology of cirrhosis:			
Viral		1.00 (Reference)	–
Alcoholic	1.23	0.42–3.62	0.71
Autoimmune	1.54	0.45–5.30	0.49
Other	1.38	0.73–2.62	0.32
Comorbid CKD	9.23	4.86–17.51	<0.001
Comorbid diabetes	2.25	1.18–4.28	0.01
Comorbid hypertension	1.4	0.65–3.38	0.36
Diuretic use	1.07	0.89–7.75	0.08
Presence of significant ascites	1.16	0.77–2.55	0.27
White blood cell count (×10⁹/L)	1.05	1.02–1.13	0.01
Neutrophils (×10⁹/L)	1.16	1.09–1.24	<0.001
Lymphocytes (×10⁹/L)	1.02	0.85–1.22	0.35
Neutrophil percentage (%)	1.05	1.03–1.08	<0.001
NLR	1.04	1.00–1.08	0.04
Hemoglobin (g/L)	0.99	0.98–1.01	0.47
Platelets (×10⁹/L)	1	0.99–1.01	0.81
ALT (U/L)	1	0.99–1.01	0.28
AST (U/L)	1	0.99–1.01	0.42
TBIL (μmol/L)	1.01	1.00–1.02	0.03
ALB (g/L)	0.91	0.87–0.97	<0.001
Blood urea nitrogen(mmol/L)	1.01	0.99–1.01	0.09
Creatinine (μmol/L)	1.01	1.00–1.01	<0.001
PT (s)	1.05	1.01–1.10	0.02
INR	0.99	0.89–1.11	0.86
TT (s)	1.041	1.00–1.08	0.03
SBP (mmHg)	0.99	0.96–1.02	0.58
DBP (mmHg)	0.99	0.95–1.03	0.59
Preoperative PPG (mmHg)	1.18	1.06–1.33	<0.001
Postoperative PPG (mmHg)	1.15	0.99–1.35	0.07
Child-Pugh score	1.34	1.09–1.63	<0.001
MELD score	1.08	1.03–1.12	<0.001

Abbreviations: AKI, acute kidney injury; ALB, albumin; ALT, alanine aminotransferase; AST, aspartate aminotransferase; CI, confidence interval; DBP, diastolic blood pressure; INR, international normalized ratio; MELD, Model for End-Stage Liver Disease; NLR, neutrophil-to-lymphocyte ratio; OR, odds ratio; PPG, portal venous pressure gradient; PT, prothrombin time; SBP, systolic blood pressure; TBIL, total bilirubin; TT, thrombin time.

**Table 3.** Univariate Cox regression to identify variables associated with the overall mortality risk.

Variables	Before PSM		After PSM	
	HR (95% CI)	*p* value	HR (95% CI)	*p* value
AKI	4.13 (2.80–6.09)	<0.001	2.92 (1.74–4.91)	<0.001
Age, yr	1.04 (1.03–1.05)	<0.001	1.02 (0.99–1.05)	0.19
Male	1.24 (0.90–1.70)	0.19	1.28 (0.74–2.22)	0.38
Etiology of cirrhosis				
Viral	1.00 (Reference)		1.00 (Reference)	
Alcoholic	1.07 (0.56–2.05)	0.83	0.62 (0.22–1.77)	0.38
Autoimmune	0.56 (0.25–1.28)	0.17	0.59 (0.21–1.69)	0.33
Other	0.91 (0.67–1.25)	0.57	0.73 (0.41–1.30)	0.29
Comorbid CKD	0.81 (0.47–1.40)	0.46	0.74 (0.34–1.64)	0.46
Comorbid diabetes	1.31 (0.88–1.95)	0.18	0.68 (0.34–1.34)	0.27
Comorbid hypertension	0.87 (0.52–1.46)	0.60	0.47 (0.22–1.00)	0.05
Comorbid hepatoma	3.41 (2.46–4.72)	<0.001	3.16 (1.62–6.18)	<0.001
Diuretic use	1.12 (0.50–2.53)	0.78	1.59 (0.75–2.26)	0.12
Presence of significant ascites	1.58 (1.16–2.16)	0.004	1.07 (0.64–1.79)	0.81
Preoperative white blood cell count (×10⁹/L)	1.04 (1.00–1.07)	0.03	1.04 (1.01–1.07)	0.01
Preoperative neutrophil count (×10⁹/L)	1.01 (0.99–1.04)	0.25	1.04 (0.98–1.10)	0.18
Neutrophil percentage (%)	1.01 (1.00–1.03)	0.02	1.02 (1.00–1.04)	0.05
Preoperative lymphocyte count (×10⁹/L)	0.74 (0.56–0.97)	0.03	0.76 (0.52–1.11)	0.16
NLR	1.03 (1.00–1.05)	0.02	1.05 (1.01–1.09)	0.009
Preoperative hemoglobin (g/L)	0.99 (0.99–1.00)	0.23	1.00 (0.99–1.01)	0.84
Preoperative platelet count (×10⁹/L)	1.00 (0.99–1.00)	0.02	1.00 (0.99–1.00)	0.61
Preoperative ALT (U/L)	1.00 (0.99–1.00)	0.33	1.00 (1.00–1.01)	0.35
Preoperative AST (U/L)	1.00 (0.99–1.00)	0.98	1.00 (1.00–1.01)	0.22
Preoperative TBIL (μmol/L)	1.01 (1.01–1.02)	<0.001	1.01 (1.00–1.02)	0.01
Preoperative ALB (g/L)	0.95 (0.93–0.97)	<0.001	0.96 (0.91–1.02)	0.18
Preoperative Blood urea nitrogen (mmol/L)	1.00 (0.99–1.01)	0.18	1.00 (1.00–1.01)	0.60
Preoperative creatinine (μmol/L)	1.00 (1.00–1.01)	0.98	1.00 (1.00–1.02)	0.40
Preoperative PT (s)	1.05 (1.03–1.07)	<0.001	1.18 (1.08–1.27)	<0.001
Preoperative INR	1.02 (1.00–1.03)	0.06	1.15 (1.03–1.39)	0.05
Preoperative TT (s)	1.03 (1.01–1.05)	0.001	1.02 (1.00–1.04)	0.04
SBP	1.01 (0.99–1.04)	0.25	1.01 (0.99–1.03)	0.23
DBP	1.02 (0.99–1.06)	0.23	1.03 (0.98–1.05)	0.26
Preoperative PPG (mmHg)	1.00 (0.95–1.05)	0.99	1.01 (0.93–1.10)	0.84
Postoperative PPG (mmHg)	0.99 (0.92–1.07)	0.92	1.13 (0.96–1.32)	0.15
Child-Pugh score	1.29 (1.18–1.44)	<0.001	1.11 (0.91–1.35)	0.31
MELD score	1.01 (1.00–1.01)	0.01	1.02 (1.00–1.03)	0.01

Abbreviations: PSM, propensity score matching; HR, hazard ratio; AKI, acute kidney injury; NLR, neutrophil-to-lymphocyte ratio; ALT, alanine aminotransferase; AST, aspartate aminotransferase; TBIL, total bilirubin; ALB, albumin; PT, prothrombin time; TT, thrombin time; INR, international normalized ratio; MELD Score, Model for End-Stage Liver Disease Score.

## Results

### Population characteristics

The patient selection process is summarized in Figure 2. Table 1 presents the baseline characteristics of the overall study population. This study retrospectively collected complete clinical data from 995 patients who underwent TIPS for portal hypertension at multiple centers between January 2015 and December 2023, including 701 (70.4%) males and 294 (29.6%) females. AKI was observed in 49 patients (4.92%), among whom 32 (65.3%) were male and 17 (34.7%) were female. Statistically significant differences (*p* < 0.05) were observed between the AKI and non-AKI groups in terms of age, use of diuretics, preoperative leukocyte count, neutrophil count, neutrophil percentage, neutrophil-to-lymphocyte ratio (NLR), blood urea nitrogen (BUN), creatinine, prothrombin time (PT), preoperative PPG, comorbid CKD, and comorbid diabetes. The incidence of post-TIPS AKI did not differ significantly among the three participating centers. Specifically, AKI occurred in 5.3% (13/246) of patients in Center 1, 4.9% (36/736) in Center 2, and 0% (0/13) in Center 3. A chi-square test confirmed no statistically significant difference in AKI rates across centers (*χ*^2^ = 0.74, *p* = 0.690). These data are presented in Table 1. Although Center 3 contributed no AKI cases, sensitivity analyses excluding this center confirmed that the identified risk factors for AKI (see Table S2) and the association between AKI and mortality (see Table S3) remained robust, indicating that the overall conclusions were not driven by data from any single center.

### Risk factor analysis for the occurrence of AKI after TIPS

A total of 995 post-TIPS patients were included in the study, with 49 patients in the AKI group, resulting in an AKI incidence of 4.92% after TIPS. Univariate analysis identified the following factors as significantly associated with the development of AKI after TIPS (*p* < 0.05): age, comorbid CKD, comorbid diabetes, leukocyte count, neutrophil count, neutrophil percentage, neutrophil-to-lymphocyte ratio, total bilirubin, albumin, creatinine, prothrombin time, thrombin time, preoperative PPG, Child-Pugh score, and MELD score (Table 2).

**Figure 1.**
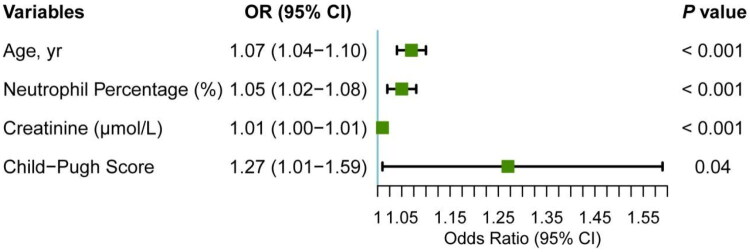
Forest plots of multivariable Logistic Regression of Risk Factors for Post-TIPS AKI. AKI, acute kidney injury; CI, confidence interval; OR, odds ratio.

Multivariable analysis revealed that age (OR: 1.07 [per years], 95%CI 1.04–1.10), preoperative neutrophil percentage (OR: 1.05 [per % change], 95% CI 1.02–1.08), creatinine (OR: 1.01 [per μmol/L], 95% CI 1.00–1.01), and Child-Pugh score (OR: 1.27, 95% CI 1.01–1.59) were independent factors associated with the development of AKI after TIPS (*p* < 0.05) (Figure 1). To verify the robustness of the study results, we performed sensitivity analyses (see Table S1), which included models with additional adjustment for comorbidities (Model 2) and substitution of the liver disease severity score (Model 3). All key risk factors remained consistently significant across these models.

**Figure 2.**
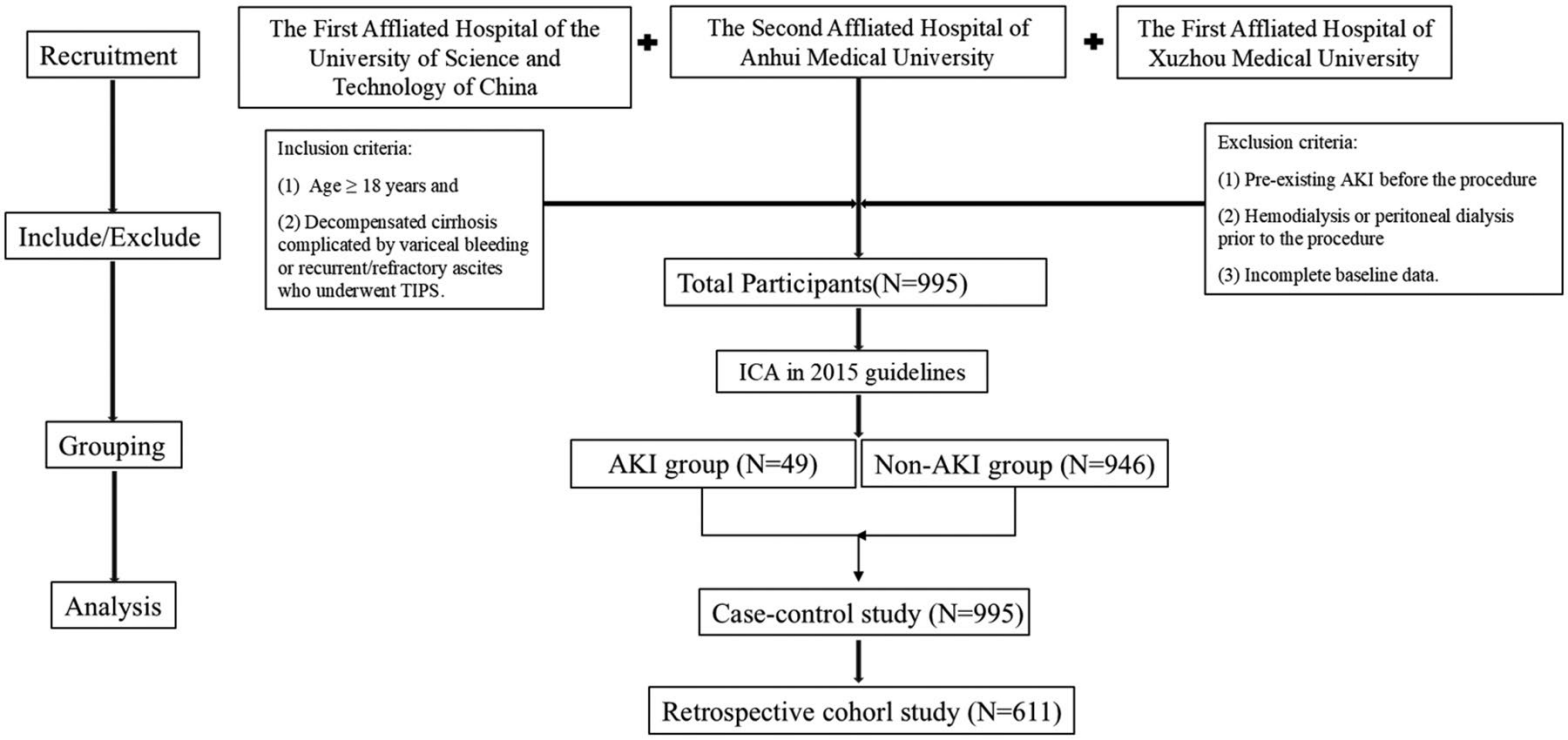
Patients selection flowchart.

### The occurrence of AKI after TIPS is associated with a poor prognosis

Among the total patients who underwent TIPS, 611 had complete medical records and follow-up data and were included in the cohort study to assess their prognosis. Of these, 414 were male and 197 were female, accounting for 67.9 and 32.1% of the cohort, respectively. A total of 46 patients developed AKI, of which 30 were male and 16 were female, representing 65.2 and 34.8% of the AKI subgroup, respectively. To minimize potential confounding, propensity score matching was performed at a 1:2 ratio, resulting in a matched cohort of 126 patients. The matched cohort comprised 42 patients in the AKI group and 84 patients in the non-AKI group. Within this cohort, 78 patients (61.9%) were male and 48 patients (38.1%) were female. The most common cause of liver disease in the AKI group compared with the non-AKI group of patients who underwent TIPS was viral hepatitis. The baseline characteristics of the cohort, both before and after matching, and comparisons between groups are shown in Table S4. Covariate balance after PSM was formally assessed using SMD, with an absolute SMD < 0.1 indicating good balance (Figure S1). Despite optimization of the matching algorithm, residual imbalance (SMD≥|0.1|) persisted for several covariates, most notably preoperative creatinine (SMD = 0.215), as well as for age, comorbid hypertension, diabetes, and hepatocellular carcinoma. In contrast, covariates including albumin, Child-Pugh score, and most etiology categories achieved good balance (SMD < |0.1|). To address the potential impact of this residual confounding, we conducted additional sensitivity Cox regression analyses within the matched cohort, adjusting for all imbalanced covariates. These analyses confirmed the significant association between AKI and mortality (Table S5).

**Figure 3.**
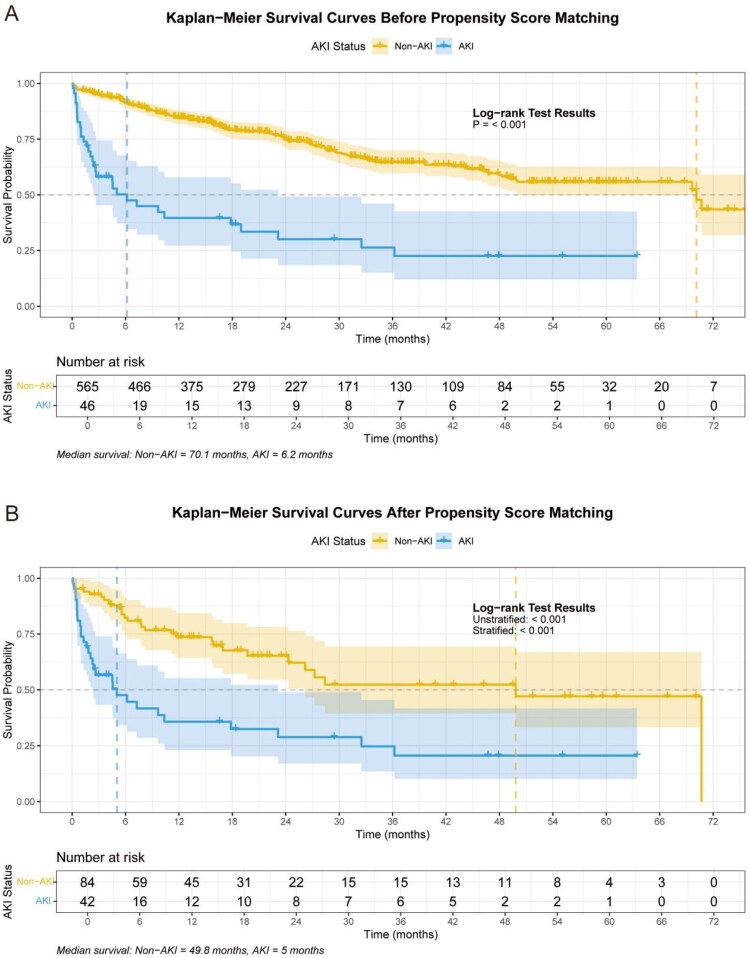
Kaplan-Meier survival curves before and after propensity score matching. (A) Overall survival in the unmatched cohort. (B) Overall survival in the propensity score-matched cohort. Survival differences were assessed using the log-rank test for the unmatched analysis and the stratified log-rank test (accounting for matched pairs) for the matched analysis. Tick marks on the curves indicate censored observations. AKI, acute kidney injury.

The occurrence of AKI was significantly associated with poor patient prognosis. In the unmatched overall cohort (Figure 3A), the median overall survival of the AKI group (6.17 months, 95% CI: 2.63–23.13) was significantly shorter than that of the non-AKI group (70.13 months, 95% CI: 50.00–NA), with a log-rank *p* < 0.001. In the propensity score-matched cohort (Figure 3B**)**, the median overall survival of the AKI group (5.03 months, 95% CI: 2.30–23.13) remained significantly shorter compared to the non-AKI group (49.83 months, 95% CI: 26.18–NA), with a stratified log-rank *p* < 0.001.

Analysis of mortality rates at key time points, as detailed in Table S4, confirmed significantly higher mortality in the AKI group both before and after propensity score matching. In the unmatched cohort, the 30-day mortality rate was 23.91% (11/46) in the AKI group versus 3.73% (21/565) in the non-AKI group (*p* < 0.05). Similarly, the 90-day (43.48 vs. 5.86%), 1-year (56.52 vs. 13.32%), 2-year (67.39 vs. 22.91%), and overall mortality (67.39 vs. 27.71%) were all markedly higher in AKI patients (all *p* < 0.05). This pattern of significantly elevated mortality associated with AKI persisted in the matched cohort.

**Figure 4.**
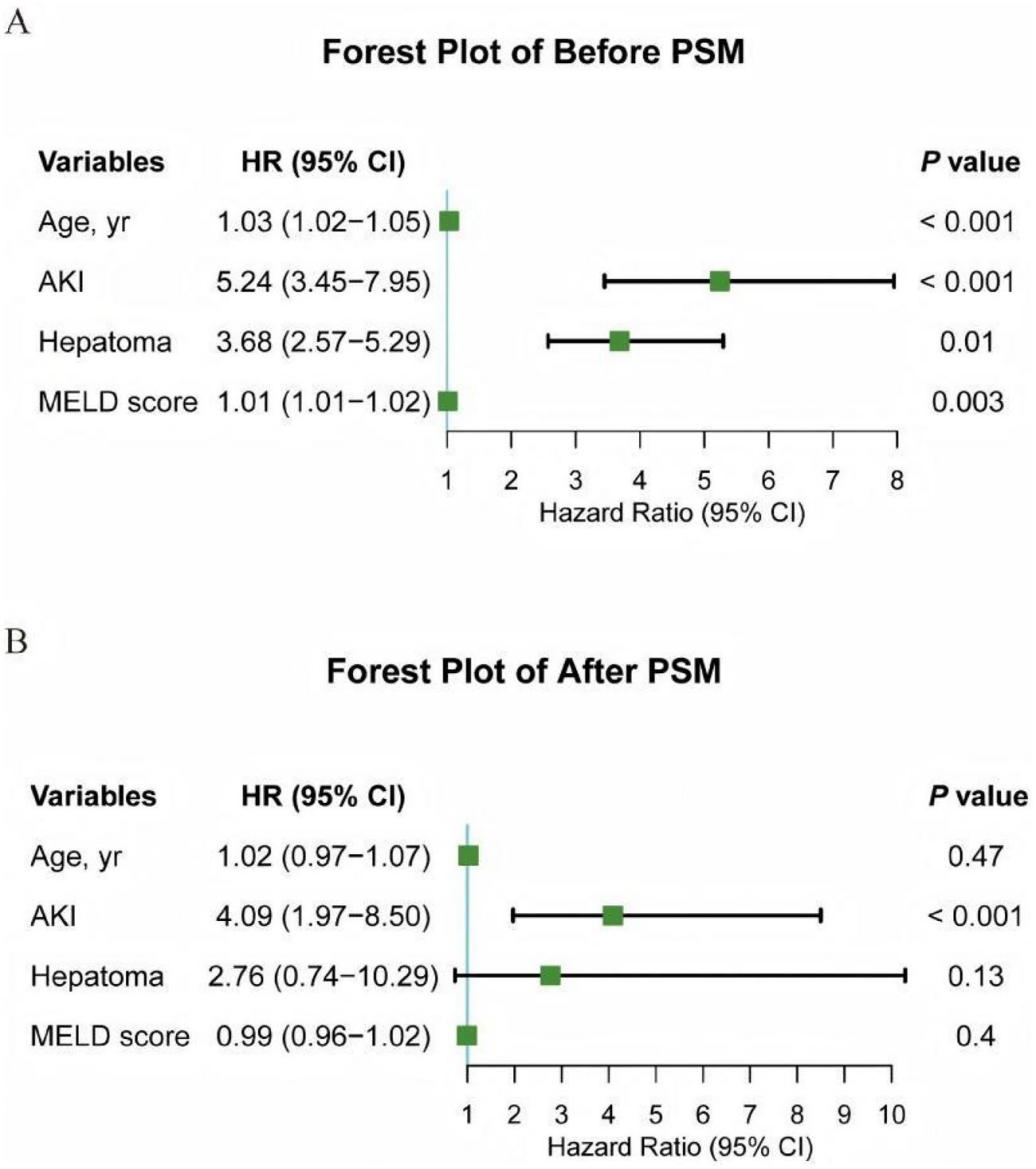
Forest plots of multivariable Cox regression analysis for mortality. (A) Before propensity score matching. (B) After propensity score matching. Variables include age, AKI, hepatoma, and MELD score.

The univariate and multivariate analysis results of the Cox proportional hazards model are shown in Table 3 and Figure 4. In the unmatched cohort, postoperative AKI, older age, presence of hepatoma, and a higher MELD score were identified as independent predictors of increased mortality (all *p* < 0.05). In the propensity score-matched cohort, stratified Cox regression analysis confirmed the strong association between AKI and mortality (HR: 4.09; 95% CI: 1.97–8.50; *p* < 0.001). This association remained robust in a sensitivity analysis where the stratified model was further adjusted for all covariates that remained imbalanced after matching (creatinine, diabetes, and hypertension) (HR for AKI: 4.36; 95% CI: 1.92–9.89; *p* < 0.001) (Table S5, Model B).

## Discussion

Our multicenter retrospective study yields several key findings regarding AKI after TIPS. The incidence of post-TIPS AKI was 4.92%, which is lower than rates reported in earlier studies such as 7.9% [7] and 16% [16]. This difference may be associated with contemporary advances in procedural technique and variations in diagnostic criteria or study populations. Historically, perioperative renal dysfunction has been a major concern in patients undergoing TIPS. Recent guidelines emphasize that renal outcomes after TIPS are closely related to baseline liver disease severity and preexisting renal impairment rather than a single procedural factor [10,17]. Our findings are consistent with this concept, highlighting the multifactorial nature of AKI development in this setting. Multivariate analysis identified older age, elevated preoperative neutrophil percentage, higher preoperative creatinine level, and an increased Child-Pugh score as independent risk factors for AKI development. These factors are consistent with established pathophysiology: advanced age is linked to reduced renal reserve [18–20], elevated neutrophil percentage reflects systemic inflammation, which has been implicated in AKI development [21,22]; higher baseline creatinine indicates preexisting renal dysfunction [23,24], and a higher Child-Pugh score reflects more severe liver disease, which can adversely affect renal perfusion and promote inflammatory responses [21]. Importantly, our study extends prior evidence, which often relied on univariate analyses [7,16], by providing an integrated, multivariate risk-assessment model.

The association between AKI and poor prognosis is notably strong. The occurrence of AKI after TIPS was linked to a markedly worse prognosis, with survival curves diverging sharply and the majority of excess mortality occurring within 90 days. This pattern aligns with previous reports of high early mortality after TIPS [25] and meta-analyses confirming the association between AKI and adverse survival outcomes [26]. Using propensity score matching, we constructed a cohort in which key indicators of liver function, including Child-Pugh score and albumin, were well-balanced between groups, enabling a more specific comparison among patients with similar baseline hepatic reserve. It is noteworthy that while matching effectively balanced liver function and several etiologies, residual imbalances persisted for age, hypertension, preoperative creatinine, diabetes, and hepatocellular carcinoma. This may reflect the inherent clinical profile of patients prone to AKI, who are often older and present with more comorbidities. Consequently, in the stratified Cox model, which rigorously adjusted for the matched-pair structure, the effects of these residually imbalanced variables (age, hepatoma) were no longer independently significant, while the association between AKI and mortality was further isolated and remained robust (HR = 4.09). This finding was further reinforced by sensitivity analysis, which demonstrated that the association between AKI and mortality persisted (HR = 4.36) even after fully adjusting for all these residually imbalanced covariates. Collectively, these results suggest that post-TIPS AKI may not merely be a transient complication but could represent a predominant and strongly independent factor associated with adverse outcomes, necessitating heightened clinical vigilance and intensified management.

This study has several strengths, including its multicenter design, which enhances generalizability, and the application of PSM alongside multivariate regression to better assess the association between AKI and mortality. However, several limitations must be acknowledged. First, a key limitation of our propensity score-matched analysis is the persistence of residual imbalance in several important baseline covariates, including preoperative creatinine, after matching. This reflects the limited overlap in baseline characteristics, particularly renal function, between patients who did and did not develop AKI, and it may influence the precision of the estimated association between AKI and mortality. Second, the retrospective design inherently limits conclusions regarding causality and may introduce unmeasured confounding; thus, the observed associations should be interpreted as correlational rather than causal effects. Third, detailed data on certain potential peri-procedural confounders, including exact contrast-agent volume, exposure to nephrotoxic medications, and standardized definitions of peri-procedural hypotension, were not uniformly available, precluding formal comparative analyses of their effects on AKI risk. Fourth, the distribution of patients and AKI events across the three participating centers was uneven, with one center contributing a very small number of patients and no AKI events. To further verify that our conclusions were not driven by the center contributing no AKI events, we repeated the core analyses after excluding data from Center 3. The results of these analyses (Supplementary Tables 2 and 3) were consistent with those from the full cohort, confirming the robustness of the identified risk factors and the AKI-mortality association. Therefore, while the uneven patient distribution across centers is noted as a limitation, its potential confounding effect has been addressed through statistical adjustment and sensitivity assessment. Fifth, although the overall cohort was sizable, the number of AKI events was limited, leading to relatively wide confidence intervals in some estimates. Finally, the study population was exclusively Chinese, with a high prevalence of hepatitis B virus-related cirrhosis; thus, the generalizability of our findings to other populations requires further validation.

In conclusion, our study identifies a distinct risk profile associated with AKI following TIPS and demonstrates a strong association between AKI and increased mortality. These findings highlight the importance of preoperative risk stratification using the identified factors age, inflammatory markers, renal function, and liver disease severity and vigilant postoperative monitoring of renal function. For patients who develop AKI after TIPS, it should be recognized as being strongly associated with poor outcomes, warranting more intensive clinical monitoring and management.

## Supplementary Material

Supplementary material.docx

## Data Availability

The datasets used during the current study are available from the corresponding author on reasonable request.
