# Supplementary material for: Incidence, associated factors and prognostic association of acute kidney injury after TIPS: a multicenter retrospective study
Source: Ren Fail. 2026 Apr 13;48(1):2650577. doi: 10.1080/0886022X.2026.2650577 (PMC13072681; doi:10.1080/0886022X.2026.2650577)
Supplement: Supplementary material.docx [file IRNF_A_2650577_SM9961.docx]

**Definition of the MELD Score**

Model for End-Stage Liver Disease (MELD) Score Calculation

The MELD score for each patient was calculated at the time of hospital admission (pre-TIPS) using the United Network for Organ Sharing (UNOS) modification of the original MELD formula (Kamath et al., Hepatology 2001;33:464-470; Wiesner et al., Gastroenterology 2003;124:91-96).

The score was derived from the following equation：

MELD = 3.78 × ln[Serum Bilirubin (mg/dL)] + 11.2 × ln[International Normalized Ratio (INR)] + 9.57 × ln[Serum Creatinine (mg/dL)] + 6.43

Where:

ln denotes the natural logarithm.

Serum Bilirubin: The total bilirubin concentration, measured in milligrams per deciliter (mg/dL).

INR: The International Normalized Ratio, a standardized measure of blood coagulation.

Serum Creatinine: The creatinine concentration, measured in mg/dL, as an indicator of renal function.

Special Considerations (UNOS Modification):

The minimum value for all three laboratory variables (Bilirubin, INR, Creatinine) was set to 1.0 for calculation purposes (i.e., any value <1.0 was entered as 1.0).

For patients undergoing renal replacement therapy (dialysis) at baseline, the serum creatinine value was set to a fixed maximum of 4.0 mg/dL in the formula [2].

The final calculated score was rounded to the nearest tenth.

Interpretation:

The MELD score is a continuous scale, with higher scores indicating greater severity of liver dysfunction and higher short-term mortality risk. In this study, it was used as a key covariate to quantify the baseline severity of liver disease.

References (within Supplementary Material):

**Kamath PS, et al. A model to predict survival in patients with end-stage liver disease. Hepatology. 2001;33(2):464-470.**

**Wiesner R, et al. Model for end-stage liver disease (MELD) and allocation of donor livers. Gastroenterology. 2003;124(1):91-96**.
